# Phylogenetic Analysis of *Massilia phlebovirus* in Portugal

**DOI:** 10.3390/v13071412

**Published:** 2021-07-20

**Authors:** Fátima Amaro, Líbia Zé-Zé, José Lourenço, Marta Giovanetti, Stefanie Christine Becker, Maria João Alves

**Affiliations:** 1 Centre for Vectors and Infectious Diseases Research, National Institute of Health Doutor Ricardo Jorge, Avenida da Liberdade n. 5, 2965-575 Águas de Moura, Portugal; libia.zeze@insa.min-saude.pt (L.Z.-Z.); m.joao.alves@insa.min-saude.pt (M.J.A.); 2 Instituto de Saúde Ambiental, Faculdade de Medicina da Universidade de Lisboa, Avenida Prof. Egas Moniz, Ed. Egas Moniz, 1649-028 Lisbon, Portugal; 3 Campus da FCUL, Biosystems and Integrative Sciences Institute, Edificio TecLabs, Campo Grande, 1749-016 Lisbon, Portugal; 4 Department of Zoology, University of Oxford, Mansfield Road, Oxford OX1 3SZ, UK; jose.lourenco@zoo.ox.ac.uk; 5 Laboratório de Flavivírus, Instituto Oswaldo Cruz Fiocruz, Avenida Brasil, Manguinhos, Rio de Janeiro 21045-900, Brazil; giovanetti.marta@gmail.com; 6 Laboratório de Genética Celular e Molecular, Universidade Federal de Minas Gerais, Avenida Antônio Carlos n. 6627, Pampula, Belo Horizonte 31270-901, Brazil; 7 Centre for Infection Medicine, Institute of Parasitology, University of Veterinary Medicine Hannover, 30559 Hannover, Germany; stefanie.becker@tiho-hannover.de

**Keywords:** phleboviruses, *Massilia phlebovirus*, Arrábida virus

## Abstract

In the last two decades, molecular surveys of arboviruses have enabled the identification of several new viruses, contributing to the knowledge of viral diversity and providing important epidemiological data regarding possible new emerging viruses. A combination of diagnostic assays, Illumina sequencing and phylogenetic inference are here used to characterize two new *Massilia phlebovirus* strains isolated from sandflies collected in the Arrábida region, Portugal. Whole genome sequence analysis enabled their identification as reassortants and the recognition of genomic variants co-circulating in Portugal. Much is still unknown about the life cycle, geographic range, evolutionary forces and public health importance of these viruses in Portugal and elsewhere, and more studies are needed.

## 1. Introduction

It is acknowledged that RNA viruses have the highest mutation and recombination rates in nature, exhibiting a remarkable capacity to evolve [1]. Among these, the ones that maintain their genomes as several distinct RNA molecules are called segmented RNA viruses. They are widespread in nature and include important human, animal and plant pathogens. A major consequence of such a genome structure is the capacity for reassortment to occur during co-infection, whereby segments may be exchanged among different co-infecting viral strains. This mechanism can create viral progeny carrying genes that are derived from more than one viral parent, providing potential evolutionary avenues for fitness and phenotypic changes to the progeny virus [2].

The genus *Phlebovirus* has been historically classified under the family Bunyaviridae, along with *Orthobunyavirus*, *Hantavirus*, *Nairovirus* and *Tospovirus* genera. In 2016, the International Committee on Taxonomy of Viruses (ICTV) created the order Bunyavirales containing nine new families, also reorganizing previously existing genera, completely replacing Bunyaviridae [3]. In this way, the genus *Phlebovirus* is now currently classified under the family Phenuiviridae. The viruses from the Phenuiviridae family are characterized by a tri-segmented negative-stranded RNA genome including a Large (L) segment (encoding the RNA-dependent RNA polymerase, RdRp), a Medium (M) segment (encoding the non-structural protein NSm and the two glycoproteins, Gn and Gc) and an ambisense Small (S) segment (encoding the nucleocapsid protein, N, and a smaller non-structural protein, NSs) [4]. According to ICTV [5], the genus *Phlebovirus* now includes a total of 66 viral species that can be found in the Old World and the Americas. The current phlebovirus species demarcation criteria consider that viruses with <95% identity in the amino acid sequence of RdRp, encoded by segment L, represent unique species.

The majority of known phleboviruses were first detected or isolated in sandflies (e.g., *Aguacate phlebovirus*, *Arbia phlebovirus*) [6,7], although there are some phleboviruses known to be circulating in other arthropods. *Rift Valley fever phlebovirus*, for example, the most important phlebovirus from a public health point of view, is transmitted mainly by mosquitoes from the genus *Aedes* and *Culex* [8]. While *Mukawa phlebovirus* has been detected in ticks, currently most tick-borne viruses formerly included in the genus *Phlebovirus* are now assigned to *Uukuvirus* genus [9,10,11].

For some viruses, e.g., *Toscana phlebovirus* (TOSV), transmitted by sandflies, the vector is also believed to be the natural reservoir and humans to be dead-end hosts, while for others such as, e.g., *Gabek phlebovirus* and *Gordil phlebovirus*, isolated from rodents, or *Anhanga phlebovirus* isolated from opossums and *Itaituba phlebovirus* from sloths, the epidemiological cycle is less clear, such that vectors and reservoirs remain to be elucidated [6,12,13,14]. TOSV is the most important sandfly-borne phlebovirus in the Mediterranean basin, since it has been reported to cause neurological disease in humans, particularly in the summer months [15]. Others, such as *Naples* or *Sicilian* phleboviruses, cause a febrile syndrome commonly known as sandfly fever in the same region [12].

In Portugal, mosquito surveillance is mandatory and nationwide, specifically on mosquitoes of the genus *Aedes* and *Culex*, which are vectors of viruses with significant relevance for Public Health, such as dengue, Zika and West Nile viruses [16,17]. Although surveillance and entomological surveys of sandflies remain scarce in the country, in surveys performed in the summers of 2007 and 2008, the isolation and the Whole Genome Sequencing (WGS) of two new phleboviruses (namely Arrábida and Alcube viruses from the Arrábida region (Setúbal county)) and the detection and WGS of two novel *Massilia phlebovirus* variants (Massilia virus isolate 127 in Arrábida region and Massilia virus isolate 130 in eastern Algarve, Olhão county) were achieved and reported [18,19].

Following results from the latter entomological surveys, in this work we present the genetic characterization of two new *Massilia phlebovirus* isolates which resulted from recent isolation attempts in sandflies from the same field collections. In addition, we introduce evidence of the co-circulation of two *Massilia phlebovirus* genomic variants in the country.

## 2. Materials and Methods

Entomological field surveys took place in the Arrábida region (Setúbal county), and eastern Algarve (Loulé, Faro, Olhão and Tavira counties), near or in animal facilities such as kennels, rabbit hutches, pigsties, chicken pens or sheep corrals, between May and October of 2007 and 2008. Sandflies were trapped with modified CDC light traps (John W. Hock Company, Gainesville, FL, USA) baited with dry ice, which were set by sunset and collected after sunrise, in periods of three consecutive nights. Alive sandflies were immediately processed or stored in dry ice or at −80 °C. The sandflies were then organized in pools, according to date, trapping origin and gender, in a maximum of 60 individuals, and macerated in liquid nitrogen. Hank’s solution (Lonza, BioWhittaker, Walkersville MD, USA) was added and the homogenate was centrifuged at 3000× *g* for 20 min, at 4 °C. PureLink Micro-to-midi Total RNA purification system (Invitrogen, Chemtrec, Carlsbad, CA, USA) was used to perform the RNA extraction. Ten percent of the collected sandflies were cleared, slide mounted and identified morphologically, according to morphological taxonomic keys [20,21].

A pan-phlebo RT-PCR assay was used for molecular screening [22]. RT-PCR positive sandfly pools homogenates were seeded in Vero E6 cell (*Cercopithecus aethiops* kidney epithelial cells, ATCC^®^ CRL 1586™) monolayer flasks for tentative isolation. Briefly, after incubation at 37 °C for one hour, Minimum Essential Medium (MEM 1X, Gibco^®^, ThermoFisher Scientific, Life Technologies Limited, Paisley, UK) supplemented with 10% fetal bovine serum (Gibco^®,^ ThermoFisher Scientific, Life Technologies Limited, Paisley, UK), and 1× Antibiotic-Antimycotic (Gibco^®^, Life Technologies Corporation, Grand Island, NY, USA) was added and the flasks were incubated at 37 °C. Flasks were examined daily for the presence of a cytopathic effect. All cultures presenting cytopathic effects were screened by RT-PCR using the above-mentioned protocol and the obtained amplicons were sequenced to confirm viral isolation, and proceeded for WGS. The samples were processed according to the protocol of Wylezich and colleagues [23] and the sequences were determined using an Illumina MiSeq (New England Biolabs, Inc., Ipswich, MA, USA), with a v3 600 cycle kit in 2 × 300 Bp paired end mode. Assembly was done with the 454/Roche assembler v3.0 with default settings. Automatically assembled sequences were analysed in silico by comparison with most similar sequences and unconfirmed projected termini (at 5′ and 3′) were removed to guarantee full length sequence quality.

Homology searches within the GenBank database were performed using the BLASTn and BLASTx algorithms [24]. Sequence alignment was performed using MAFFT (FFT-NS-2 algorithm) and visually inspected in AliView [25,26]. Phylogenetic analysis was performed using RdRp amino acid (*n* = 27) and total concatenated nucleotide (*n* = 13) sequences of genome segments, which were retrieved from GenBank (alignments available in Supplementary Material). We estimated maximum-likelihood phylogenies in IQ-TREE [27] by using the best-fit model of nucleotide substitution as indicated by the ModelFinder application (implemented in IQ-TREE), while 1000 ultrafast bootstrap replicates and SH-aLRT tests were applied to evaluate the robustness of the inferred tree. Phylogenetic trees were edited in figtree v1.44 (http://tree.bio.ed.ac.uk/software/figtree/, accessed on 8 June 2021), and genetic distance matrices between amino acid alignments of RdRp and glycoprotein precursor coding regions obtained from MegaX were plotted using in house R scripts (v4.02) based on ggplot2 [28]. Sequence data used in the alignments are depicted in Table S1: Phleboviruses sequence data. The used sequence alignments can be accessed as supplementary fasta files (File S1: Phleboviruses’ amino acid sequence alignment of L segments and File S2: Phleboviruses’ nucleotide concatenated sequence alignment of all segments). Since the presentation of concatenated trees is not common in the literature, phylogenetic trees of amino acid glycoprotein precursor (segment M) and N and NSs proteins (segment S) were also inferred, and are available for comparison in Supplementary File S3: Additional Phleboviruses’ trees.

Finally, the alignment of concatenated whole-genome sequences (L + M + S) of 13 phlebovirus (with more than 70% pairwise sequence identity), also used for phylogenetic analysis, was screened for recombination using Recombination Detection Program (RDP) v4.101 using default settings [29]. RDP implements analysis of recombination using seven different detection algorithms: RDP [30], GENECONV [31], BootScan [32], MaxChi [33], Chimaera [34], SiScan [35] and 3Seq [36]. Only recombinant sequences detected by at least five implemented algorithms and with *p*-values of <1.0 × 10^−6^ were further analyzed as positive/possible recombinant events [29].

## 3. Results

A total of 6137 sandflies divided in 237 pools (110 from Algarve counties and 127 from Setúbal county) were screened. In the eastern region of Algarve four sandfly species were found: *Phlebotomus perniciosus*, *Ph. Sergenti*, *Ph. ariasi* and *Sergentomyia minuta* (91%, 3%, 3%, 3%, respectively). In the Arrábida region, three sandfly species were identified: *Ph. perniciosus*, *Ph. sergenti* and *Ph. ariasi* (94%, 4% and 2% respectively).

Isolation attempts from different sandfly pools were performed and cytopathic effect was visible on the fourth day after infection, with two new isolates obtained, here named PoSFPhlebV/21/2007 and PoSFPhlebV/70/2007. These two pools were composed of 50 and nine sandfly females, respectively, collected in two different farms in the Arrábida region. Sandfly specimens from the sample pools were morphologically identified as *Ph. perniciosus*.

**Figure 1:**
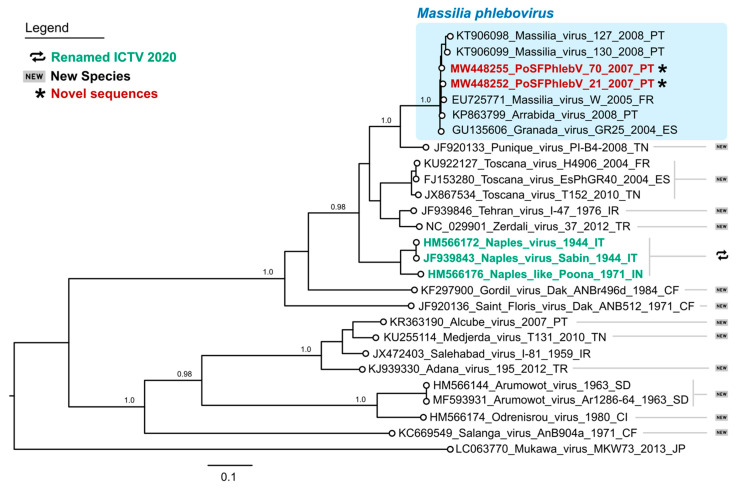
Maximum likelihood phylogenetic tree of phleboviruses entire RNA-dependent RNA polymerase protein sequences. Sequence names in red and marked with an asterisk have been sequenced in this study. According to the International Committee on Taxonomy of Viruses 2020, sequence names in green have recently had their species renamed, while those marked by “new” symbols on the right side of the tree have been assigned as new species. Names of the sequences in the tree match the IDs in the alignment made available as a supplementary file. Numbers on branches are the bootstrap support. The shaded area on the top highlights the Massilia phlebovirus cluster. Used sequence alignment is available in supplementary File S1: Phleboviruses’ amino acid sequence alignment of L segments.

**Figure 2:**
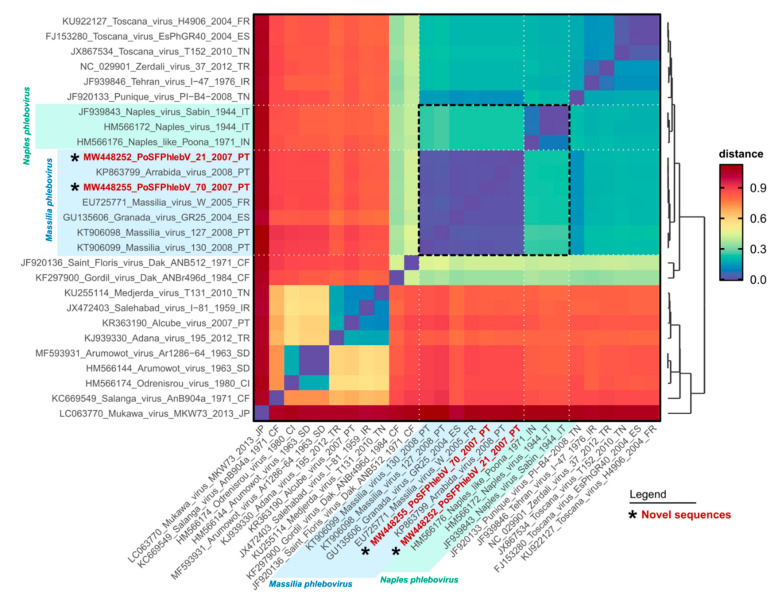
Pairwise genetic distance heatmap of phleboviruses RNA-dependent RNA polymerase protein sequences. Each cell shows the genetic distance between pairs of sequences (*y* versus *x* axis), colored according to the scale presented on the right. The dendrogram on the right was built using the pairwise distances with the base function hclust in R, and is reflective of the named sequences on the y axis. Sequence names in red and marked with an asterisk have been sequenced in this study. The shaded areas on the axes highlight the classic Massilia phlebovirus group (blue) and the newly renamed Naples phlebovirus group (green). The dashed black square in the heatmap highlights the distance results for the sequences of the Massilia and Naples groups. The sequences used for this output are the same presented in Figure 1. Original pairwise distance data is presented in Table S2: Estimates of evolutionary divergence between phleboviruses’ RdRp aa sequences.

The WGS analysis displayed the characteristic tri-segmented genome organization of phleboviruses. The complete sequence of PoSFPhlebV/21/2007 included 1854 nt, 4221 nt and 6404 nt for the S, M and L segments, respectively (GenBank acc. no. MW448250-MW448252), and PoSFPhlebV/70/2007 included 1873 nt, 4229 nt and 6386 nt (GenBank acc. no. MW448253-MW448255). The phylogenetic analyses based on the RdRp proteins showed that these two new isolates can be assigned to the *Massilia phlebovirus* species, together with other *Massilia phlebovirus* variants and Arrábida and Granada viruses. Interestingly, our analysis regarding Arrábida and Granada viruses species assignment conflicts with the ICTV inclusion of these viruses in *Naples phlebovirus* species (Figure 1 and Figure 2, and Table S2: Estimates of evolutionary divergence between phleboviruses’ RdRp aa sequences).

**Figure 3:**
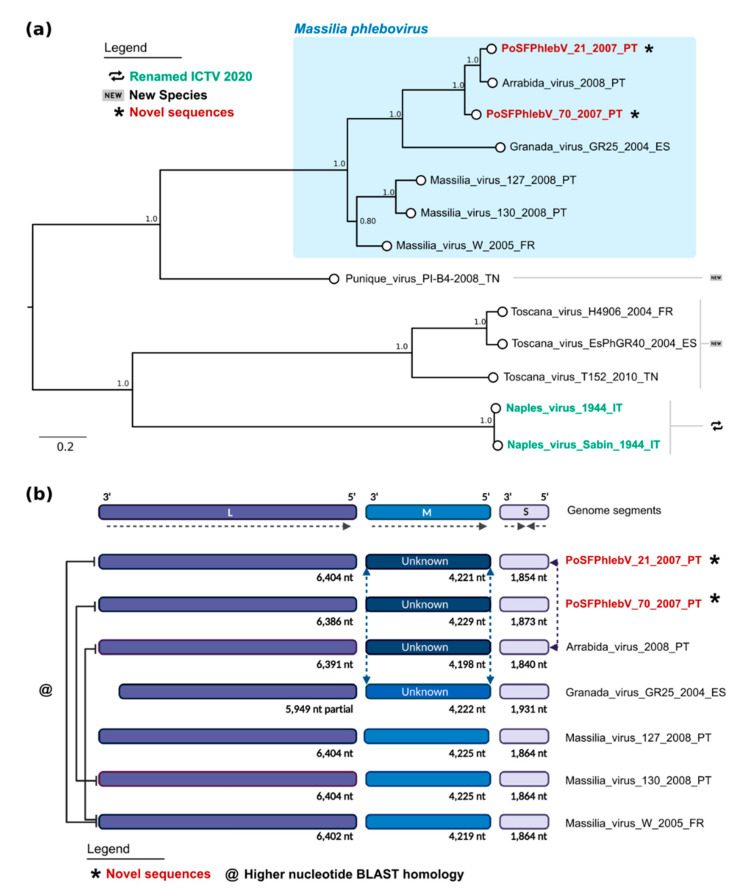
Maximum likelihood phylogenetic tree of phleboviruses’ full nucleotide concatenated segments and recombination results. (**a**) Phylogenetic tree. Sequence names in red and marked with an asterisk have been sequenced in this study. According to the International Committee on Taxonomy of Viruses 2020, sequence names in green have recently had their species renamed, while those marked by “new” symbols on the right side of the tree have been assigned as new species. Names of the sequences in the tree match the IDs in the alignment made available as a supplementary File S2: Phleboviruses’ nucleotide concatenated sequence alignment of all segments. Numbers on branches are the bootstrap support. The shaded area on the top shows the classic Massilia phlebovirus group. (**b**) Schematic of genome data and recombination results from RDP (created with https://biorender.com/). The full lines on the left link the genomes with highest nucleotide homology in the segment L sequence.

The maximum likelihood phylogenetic tree of phleboviruses’ full nucleotide concatenated segments showed that the two new strains group with the previously described Arrábida and Granada viruses with maximum statistical support (bootstrap = 100%, Figure 3a). The two already reported Portuguese Massilia strains, including the one detected in Arrábida region (Massilia virus 127) and the other in Olhão county, eastern Algarve (Massilia virus 130), appear to be more closely related with the Massilia virus W strain, isolated in France. This seemingly separation into two clusters was also evident by Blast analysis with segment M and pairwise distances within glycoprotein precursor sequences (Table S3: Estimates of evolutionary divergence between phleboviruses glycoprotein precursor sequences).

This analysis and the previous reassortment events reported in Granada and Arrábida viruses suggest the possibility of similar reassortment events in PoSFPhlebV/21/2007 and PoSFPhlebV/70/2007 [18,37].

RDP analysis using full genome concatenated sequences (segments L + M + S) sequences confirmed recombination events in segment M of the two new Massilia viruses PoSFPhlebV/21/2007 and PoSFPhlebV/70/2007 aside with Arrábida virus (Figure 3b). These events involve an unknown virus more closely related to Punique virus and show high support in RDP analysis (Figure S1: Schematic analysis of RDP most supported recombinant results). In PoSFPhlebV/21/2007 M segment reassortment is assigned with segment S, expressing a higher similarity to the Arrábida virus segment S sequence (Figure 3b and Figure S1). This result agrees with the clusters obtained from whole genome analysis (Figure 3a). Three additional recombination events (less supported but within the defined parameters) were also detected by RDP, however possible misidentification of the recombinant is considered, and as such they were no further considered. More studies on phleboviruses’ diversity are needed to clarify the importance of recombination/reassortment in their current phylogenetic structure and evolution.

Whole genome data analysis confirms the circulation of two *Massilia phlebovirus* genomic variants. One including PoSFPhlebV/21/2007 and PoSFPhlebV/70/2007 strains, Arrábida and Granada viruses, and the second including Massilia virus strain W from France and the ones previously isolated in Portugal [18,19,38]. These variants are mainly defined by segment M divergency, probably related to an ancient reassortment event associated with the first variant. Blast analysis (BlastN and BlastX) also confirm these results (Figure 3 and Table S3: Estimates of evolutionary divergence between phleboviruses glycoprotein precursor sequences).

## 4. Discussion

As with many known viruses (e.g., flaviviruses), species of phleboviruses were initially defined through serological inference, based on cross-reactivity or neutralization assays [39]. With the advent of molecular techniques, WGS and identification of an ever-growing number of species, however, such criteria are no longer the standard [6]. The ICTV now proposes that viral genomes with <95% amino acid sequence identity in RdRp represent unique species, which is the current criteria for the distinction of 66 known species [5]. According also to ICTV, the Arrábida and Granada viruses are included in *Naples phlebovirus* species [10]. However, considering the current ICTV definition of *Phlebovirus* species, the new strains reported here and, similarly, Granada and Arrábida viruses should be assigned to *Massilia phlebovirus* species, along with other *Massilia phlebovirus* strains.

Given the segmented nature of these viruses, co-infection with related lineages can result in genetic reassortment. In other words, if two closely related viruses coinfect the same host or cell, it is possible that RNA segments from either of the parental viruses will be incorporated into progeny virions to give reassortant viruses [40]. Co-circulation of viruses in the same geographic region increases the probability of co-infection of vectors and/or hosts, which is essential to produce reassortant viruses in nature. Particularly relevant is the apparent frequency of reassortment events in M segments, in which the S and L segments of a parent virus and the M segment of an heterologous virus can be combined to generate suitable progeny [6,18,37,40]. To date, most recognized bunyavirus reassortants have been described as following this sort of arrangement [6,41]. In these viruses, reassortment has seemingly occurred both between distant (intertypic) and closely-related (intratypic) lineages [41,42]. As in the population biology of other viruses (e.g., Influenza A), reassortment is a mechanism of diversification and adaptation [43], with potential implications for fitness, pathogenicity and host-range [44]. In general, reassortants pose challenges to the current definition of species as per the ICTV criteria, highlighting the possibility that more general considerations may be required for deciding under what conditions particular reassortants should be considered as members of an existing or new species.

In this work, we present a focused phylogenetic approach, analyzing the concatenated genome of a small group of closely related phleboviruses’ sequences (13 sequences with more than 70% pairwise sequence identity) and, in this way, integrating all genome signal information. Since the L and S segments are clearly less divergent than the M segment (that codes for antigenic traits), analysis including all genome data should provide a more realistic perspective. The resulting phylogenetic tree using full nucleotide concatenated sequences showed two main clusters within *Massilia phlebovirus* not defined using solely L segment data, in agreement with recombination analysis and detected genome features. Although this approach may work using a small cluster of related sequences, a global analysis within the genus with the addition of more divergent genome sequences should be addressed carefully, since the obtained alignments lose robustness. These two *Massilia phlebovirus* clusters are curiously also differentiated in recombination analysis, since the sequences with a detected recombinant event in S (strains 127, 130 and W; minor parent Arrábida virus, less supported event) are the variants that do not show reassorment of segment M, suggesting a common ancestor sharing these features for each cluster. Both Massilia phlebovirus genomic variants were detected in the south of Portugal, however a more expanded geographic range is possible but unknown.

Following this line of reasoning, to date, phleboviruses included in the *Massilia phlebovirus* species have been isolated in sandflies from France, Spain and Portugal. Its public health importance is largely unknown, but it is now clear that it is at least associated with the vector *Ph. perniciosus* since the first isolation was made from one male *Ph. perniciosus* [38]. Although seropositivity for Granada virus has been found in the local human population, there is, to date, no demonstration that this group of viruses can cause symptomatic infections [37,45]. Furthermore, studies are needed for the identification of potential vertebrate hosts of *Massilia phlebovirus* beyond humans in order to clarify its ecological cycle. There are examples of sandfly-borne viruses initially isolated from sandflies which were then shown to be pathogenic to humans, such as TOSV which was isolated in 1971 and only associated with human disease in 1983, and Adria virus, isolated from sandflies collected in 2005 and reported as a human pathogen due to its detection in a febrile child in 2011 [7,46,47,48]. The possibility that a wider number of unidentified but already circulating viruses from this group could be of public health importance should also not be neglected.

The Arrábida region is located in the southern coastline of Lisbon (40 km away) and involves a protected mountain chain area with typical Mediterranean vegetation. So far, five phleboviruses with specific sequence signatures were detected in the Arrábida region, namely, Arrábida virus, these two new reported isolates (comprising one *Massilia phlebovirus* genomic variant), Massilia 127 (member of the other *Massilia phlebovirus* genomic variant) and a more divergent phlebovirus recently recognized as a new species, *Alcube phlebovirus*. Four of these viruses were detected in the same collection site, indicating that this region may be a particular hotspot to study phleboviruses’ diversity.

## Data Availability

All data used and presented in this study is either available in public repositories as described in the Methods section, or is made available in Supplementary Files with this article.

## Supplementary Materials

The following are available online at https://www.mdpi.com/article/10.3390/v13071412/s1, Table S1: Phleboviruses’ sequence data, Table S2: Estimates of evolutionary divergence between phleboviruses’ RdRp aa sequences, Table S3: Estimates of evolutionary divergence between phleboviruses glycoprotein precursor sequences, Figure S1: Schematic analysis of RDP most supported recombinant results, File S1: Phleboviruses’ amino acid sequence alignment of L segments, File S2: Phleboviruses’ nucleotide concatenated sequence alignment of all segments, File S3: Additional Phleboviruses’ trees.
