# Supplementary material for: Phylogenetic Analysis of Massilia phlebovirus in Portugal
Source: Viruses. 2021 Jul 20;13(7):1412. doi: 10.3390/v13071412 (PMC8310352; doi:10.3390/v13071412)
Supplement: Supplementary file 1 [file viruses-13-01412-s001.zip › File_S3_Additional Phlebovirusesí» trees.pdf]

Supplementary File S3: Additional Phleboviruses' trees

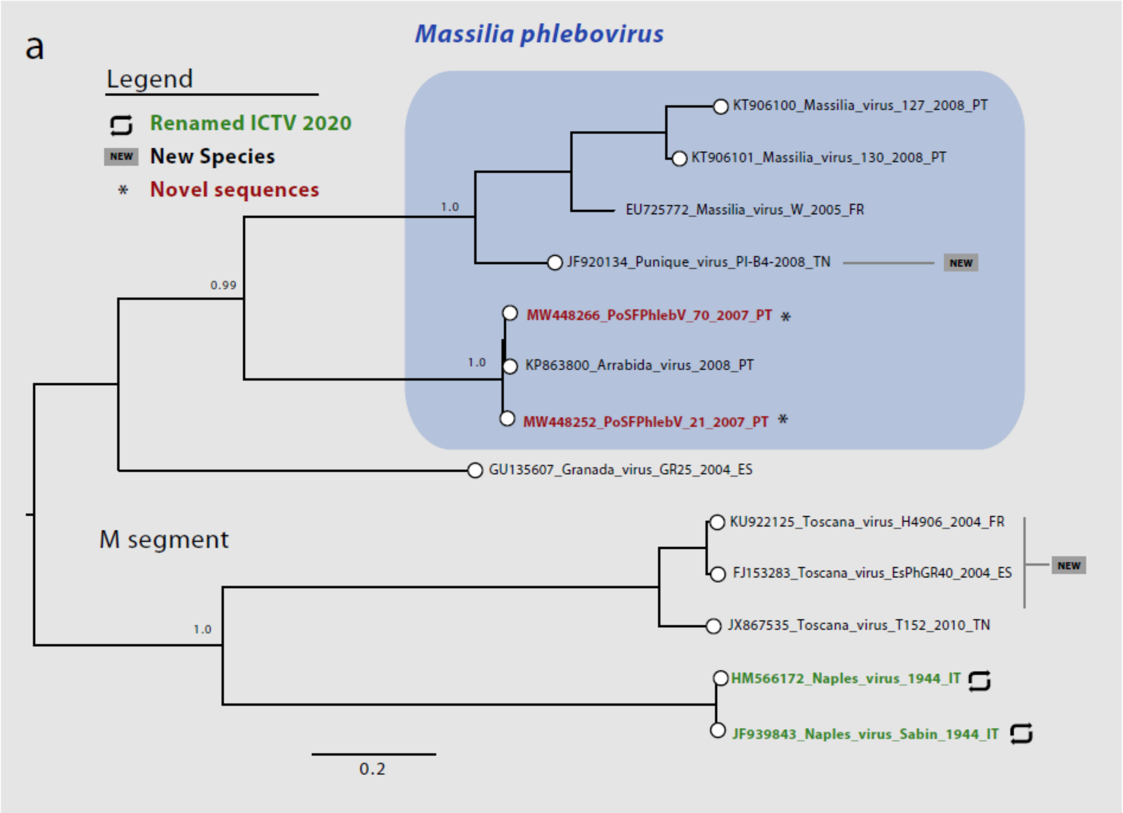

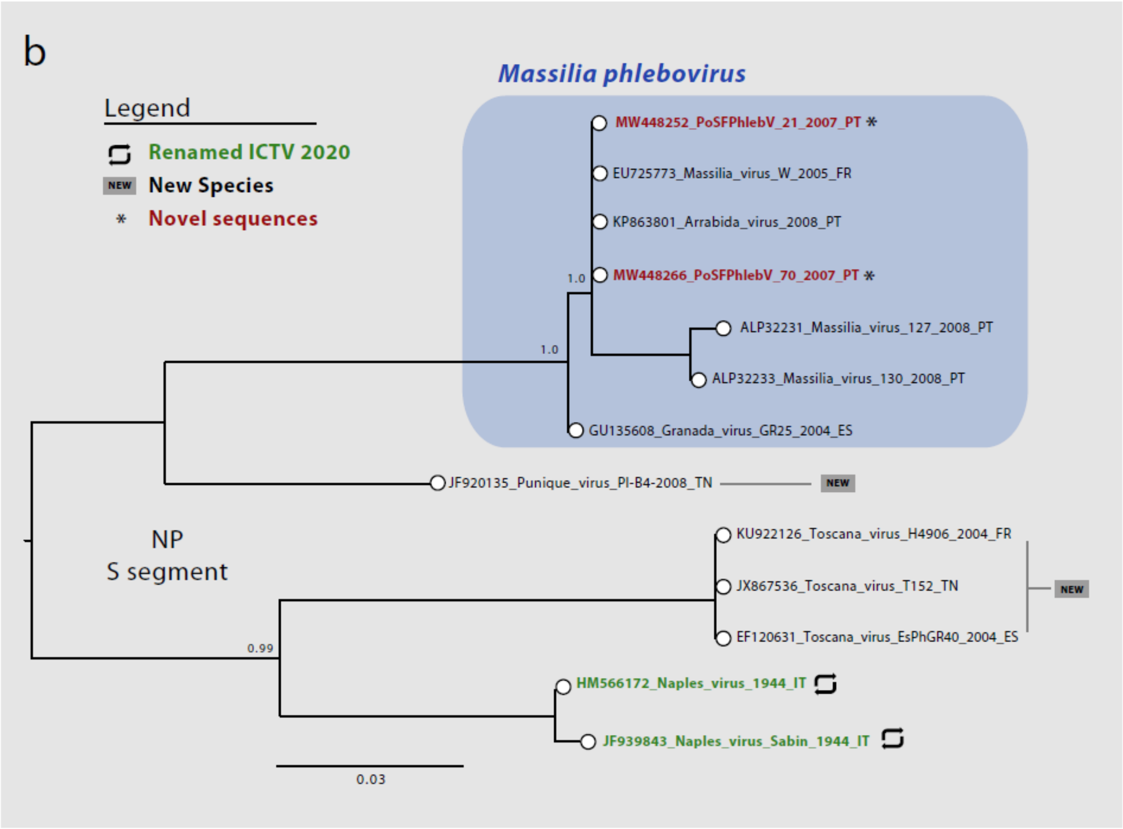

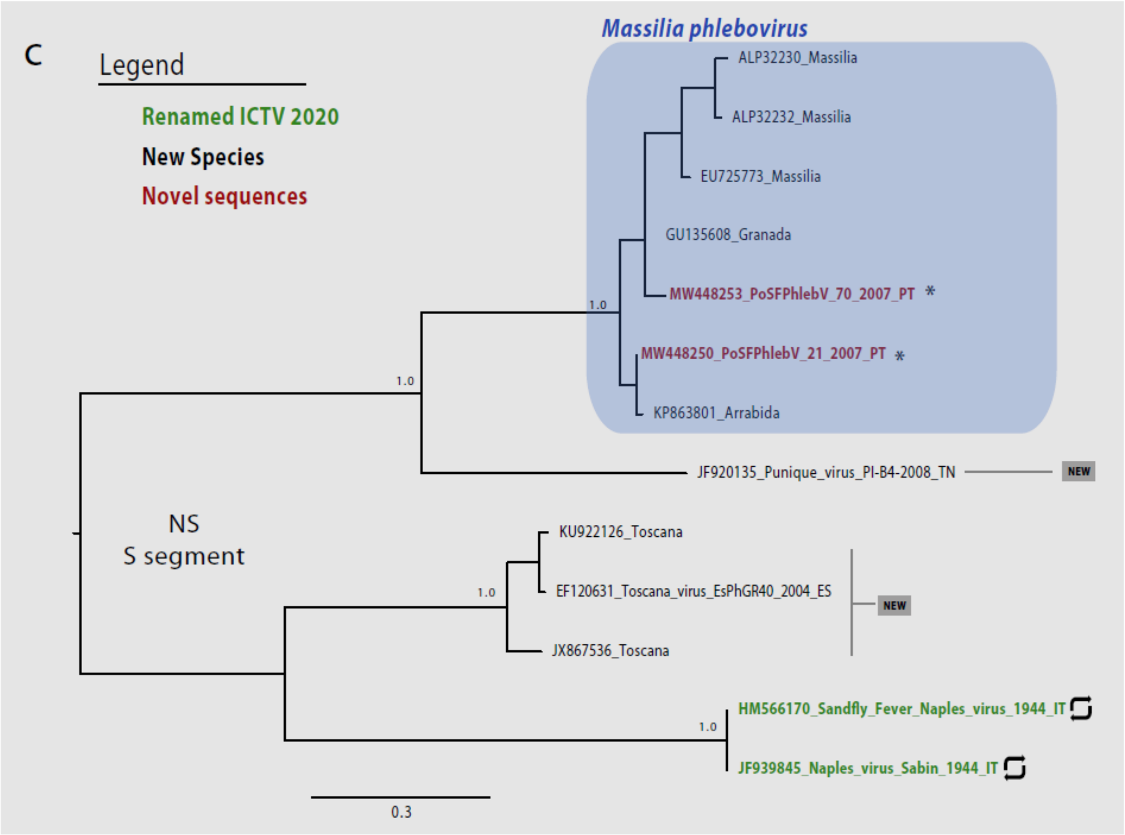

__Supplementary File S3: Additional Phleboviruses' trees__

Maximum likelihood phylogenetic tree of phleboviruses entire amino acid sequences of: a. Glycoprotein percursor protein (segment M); b. Nucleoprotein (NP, segment S); c. Non-structural protein (NS, segment S). Sequence names in red and marked with an asterisk have been sequenced in this study. According to ICTV 2020, sequence names in green have recently had their species renamed, while those marked by "new" symbols on the right side of the tree have been assigned as new species. Names of the sequences in the tree match the IDs in the alignment made available as a supplementary file. Numbers on branches are the bootstrap support. The shaded area on the top highlights the Massilia phlebovirus cluster (please note that in a., showing the phylogenetic tree based in segment M sequences, *Punique phlebovirus* is included within Massilia phlebovirus cluster due to reassortment events).
