# Supplementary material for: Phylogenetic Analysis of Massilia phlebovirus in Portugal
Source: Viruses. 2021 Jul 20;13(7):1412. doi: 10.3390/v13071412 (PMC8310352; doi:10.3390/v13071412)
Supplement: Supplementary file 1 [file viruses-13-01412-s001.zip › Headings_or_Titles_Supplementary_materials.pdf]

Headings or Titles

Table S1: Phleboviruses' sequence data

Table S2: Estimates of evolutionary divergence between phleboviruses' RdRp amino acid sequences

Table S3: Estimates of evolutionary divergence between phleboviruses' glycoprotein precursor sequences

Figure S1: Schematic analysis of RDP most supported recombinant results

File S1: Phleboviruses' amino acid sequence alignment of L segments

File S2: Phleboviruses' nucleotide concatenated sequence alignment of all segments
