# Supplementary material for: Phylogenetic Analysis of Massilia phlebovirus in Portugal
Source: Viruses. 2021 Jul 20;13(7):1412. doi: 10.3390/v13071412 (PMC8310352; doi:10.3390/v13071412)
Supplement: Supplementary file 1 [file viruses-13-01412-s001.zip › Table_S1_Sequence_data.pdf]

Table S1. Phleboviruses' sequence data

Sample ID	Species designation ICTV2020[¶]	GenBank Accession L Seg	Country first isolated/detected	Year	Isolation Source 1	Isolation Source 2	Reference
Massilia PoSFPhlebV/21/2007	*Massilia phlebovirus*	MW448252	Portugal	2007	Sandflies	Phlebotomine sandflies	This work
Massilia PoSFPhlebV/70/2007	*Massilia phlebovirus*	MW448255	Portugal	2007	Sandflies	Phlebotomine sandflies	This work
Arrabida virus	*Naples phlebovirus**	KP863799	Portugal	2008	Sandflies	Phlebotomine sandflies	DOI: 10.1016/j.virusres.2016.01.004
Massilia_127	*Massilia phlebovirus*	KT906098	Portugal	2008	Sandflies	Phlebotomine sandflies	DOI: 10.1186/s12985-015-0407-0
Massilia_130	*Massilia phlebovirus*	KT906099	Portugal	2008	Sandflies	Phlebotomine sandflies	DOI: 10.1186/s12985-015-0407-0
Massilia_W	*Massilia phlebovirus*	EU725771	France	2005	Sandflies	*Phlebotomus perniciosus*	DOI: 10.1089/vbz.2008.0131
Granada_GR25	*Naples phlebovirus**	GU135606	Spain	2004	Sandflies	*Phlebotomine sandflies*	DOI: 10.4269/ajtmh.2010.09-0697
Punique_PI_B4	*Punique phlebovirus*	JF920133	Tunisia	2008	Sandflies	*Ph. perniciousus, Ph. longicuspis*	DOI: 10.1099/vir.0.056614-0
Toscana_H4906	*Toscana phlebovirus*	KU922127	France	2004	Human	*Homo sapiens*	DOI: 10.1128/genomeA.00454-16
Toscana_T152	*Toscana phlebovirus*	JX867534	Tunisia	2010	Sandflies	*Phlebotomus* sp.	DOI: 10.3201/eid1902.121463
Toscana_EsPHGR40	*Toscana phlebovirus*	FJ153280	Spain	2004	Sandflies	*Phlebotomus* sp.	DOI: 10.3201/eid1504.081111
Sandfly_Fever_Naples	*Naples phlebovirus*	HM566172	Italy	1944	Human	*Homo sapiens*	
Naples_Sabin	*Naples phlebovirus*	JF939843	Italy	1944	Human	*Homo sapiens*	DOI: 10.1099/vir.0.056614-0
Sand_fever_Naples_like_strain Poona	*Naples phlebovirus*	HM566176	India	1971	Human	*Homo sapiens*	
Gordil_virus_isolate_Dak_ANBr 496d	*Gordil phlebovirus*	KF297900	Central African Republic	1984	Mouse	*Lemniscomys striatus*	DOI: 10.1099/vir.0.056614-0
Tehran_virus_strain_I-47	*Tehran phlebovirus*	JF939846	Iran	1976	Sandflies	*Phlebotomus perfiliewi*	DOI: 10.1099/vir.0.056614-0
Saint_Floris_virus_strain_Dak_ANB 512	*Saint Floris phlebovirus*	JF920136	Central African Republic	1971	Mouse	*Tatera* sp.	DOI: 10.1099/vir.0.056614-0
Mukawa_virus_isolate_MKW73	*Mukawa phlebovirus*	LC063770	Japan	2013	Tick	*Ixodes persulcatus*	DOI: 10.1128/mSphere.00239-18
Zerdali_virus_strain_37	*Zerdali phlebovirus*	NC_029901	Turkey	2012	Sandflies	*Phlebotomus* sp.	DOI: 10.1371/journal.pntd.0004519
Alcube_virus_S20	*Alcube phlebovirus*	KR363190	Portugal	2007	Sandflies	*Phlebotomine sandflies*	DOI: 10.1186/s12985-015-0407-0
Medjerda_Valley_virus_strain_T131	*Medjerda phlebovirus*	KU255114	Tunisia	2010	Sandflies	*Phlebotomus* sp.	DOI: 10.1099/jgv.0.000389
Salehabad_virus_strain_I-81	*Salehabad phlebovirus*	JX472403	Iran	1959	Sandflies	*Phlebotomus* sp.	DOI: 10.1099/vir.0.048850-0
Adana_virus_strain_195	*Adana phlebovirus*	KJ939330	Turkey	2012	Sandflies	*Phlebotomus* sp.	DOI: 10.1128/JVI.03027-14
Arumowot_virus	*Arumowot phlebovirus*	HM566144	Sudan	1963	Mosquitoes	*Culex antennatus*	DOI: 10.1099/vir.0.048850-0
Arumowot_virus_isolate_Ar_1286-64	*Arumowot phlebovirus*	MF593931	Sudan	1963	Mosquitoes	*Culex antennatus*	DOI: 10.1371/journal.pone.0185194
Odrenisrou_virus	*Odrenisrou phlebovirus*	HM566174	Ivory Coast	1980	Mosquitoes	*Culex albiventris*	DOI: 10.1099/vir.0.048850-0
Salanga_virus_strain_AnB_904a	*Salanga phlebovirus*	KC669549	Central African Republic	1971	Rodent	*Aethomys medicatus*	DOI: 10.1371/journal.pone.0078470

[¶] Archives of Virology (2020) 165:3023–3072; DOI:10.1007/s00705-020-04731-2

Reference and Genbank data differ. The data presented was retrieved from Genbank accession.

Isolation Source 1 - common species/group name; **Isolation Source 2 -** species assigned
