# Supplementary figures and images for: Phylogenetic Analysis of Massilia phlebovirus in Portugal
Source: Viruses. 2021 Jul 20;13(7):1412. doi: 10.3390/v13071412 (PMC8310352; doi:10.3390/v13071412)

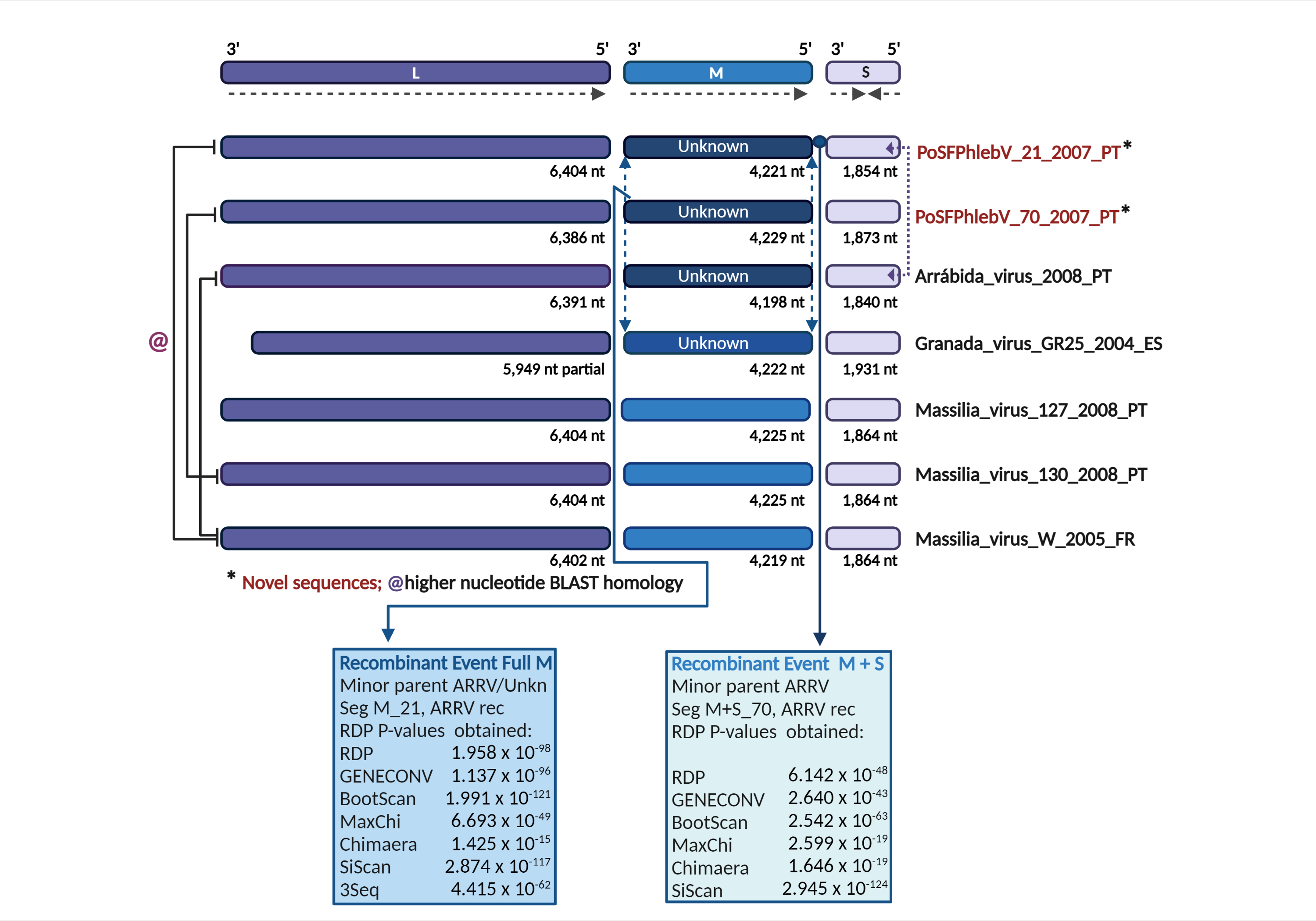

Supplement: Supplementary file 1 [file viruses-13-01412-s001.zip › Figure_S1_Schematic_analysis_RDP.jpeg]
